# Vital Sign Monitoring and Mobile Phone Usage Detection Using IR-UWB Radar for Intended Use in Car Crash Prevention

**DOI:** 10.3390/s17061240

**Published:** 2017-05-30

**Authors:** Seong Kyu Leem, Faheem Khan, Sung Ho Cho

**Affiliations:** Department of Electronics and Computer Engineering, Hanyang University, 222 Wangsimini-ro, Seongdong-gu, Seoul 04763, Korea; cromy07@hanyang.ac.kr (S.K.L.); faheemkhan@hanyang.ac.kr (F.K.)

**Keywords:** collision prevention, car safety, driver behavior, drowsiness, distraction, vital signs, IR-UWB radar, phone detection

## Abstract

In order to avoid car crashes, active safety systems are becoming more and more important. Many crashes are caused due to driver drowsiness or mobile phone usage. Detecting the drowsiness of the driver is very important for the safety of a car. Monitoring of vital signs such as respiration rate and heart rate is important to determine the occurrence of driver drowsiness. In this paper, robust vital signs monitoring through impulse radio ultra-wideband (IR-UWB) radar is discussed. We propose a new algorithm that can estimate the vital signs even if there is motion caused by the driving activities. We analyzed the whole fast time vital detection region and found the signals at those fast time locations that have useful information related to the vital signals. We segmented those signals into sub-signals and then constructed the desired vital signal using the correlation method. In this way, the vital signs of the driver can be monitored noninvasively, which can be used by researchers to detect the drowsiness of the driver which is related to the vital signs i.e., respiration and heart rate. In addition, texting on a mobile phone during driving may cause visual, manual or cognitive distraction of the driver. In order to reduce accidents caused by a distracted driver, we proposed an algorithm that can detect perfectly a driver's mobile phone usage even if there are various motions of the driver in the car or changes in background objects. These novel techniques, which monitor vital signs associated with drowsiness and detect phone usage before a driver makes a mistake, may be very helpful in developing techniques for preventing a car crash.

## 1. Introduction

Every year, according to the statistics, more than a million people die on the world’s roads due to car crashes and the cost of dealing with the consequences of these crashes runs to billions [1]. Researchers are trying to reduce these numbers by employing different techniques [2,3,4,5,6,7,8]. Many people are saved during collisions by air bags, seat belts, and the car frames that can absorb a huge amount of energy during a car crash [9]. However, the ultimate solution is to keep cars from smashing into each other in the first place. Adaptive cruise control (ACC) systems use laser beams or radar to measure the distance between the cars and their relative speed [10]. All of the ACC systems available today are built around sensors that detect the vehicle ahead with either radar or light detecting and ranging (LIDAR), the laser-based analog to radar [11]. These systems do detect and prevent some types of driver error. However, technologies cannot detect and prevent all of the mistakes that drivers make and can result in collisions. The major driver mistakes are caused by drowsiness [12] and mobile phone usage during driving.

According to a recent study, 20% of crashes occurred due to drowsiness of the driver [13]. Continuous monotonous driving for a long time causes drowsiness, which may result in fatal road accidents. For long haul/distance drivers, fatigue and drowsiness can hardly be avoided [13]. Thus, research works are carried out to investigate approaches to monitor driver condition. Various methods are used to detect if a driver is tired or falling asleep. Researchers have attempted to determine driver drowsiness using the following measures: (1) vehicle-based measures [14,15] (2) behavioral measures [16,17,18] and (3) physiological measures [19,20,21,22,23]. Mercedes-Benz pioneered one of the first techniques, which uses a computer algorithm that compares the steering behavior of a driver to the trained behavior at the start of the trip [9]. Other systems monitor the location of car within its lane of travel, looking for erratic maneuvers indicative of inattention. Some researchers also track the driver eye movement with an in-car camera, noting rapid or prolonged eye blinks. In [14,15], the researchers used metrics like lane position, steering wheel position, driving speed and car yaw angle for detecting the drowsiness of drivers. These metrics are useful, but this method is an alarm for the mistake after the happening, so it cannot prevent in advance the potential mistakes caused by drowsiness. An eye-tracking based scheme is proposed for detection of driver drowsiness using an unscented Kalman filter [16]. In reference [17], the researchers used facial image sequences to detect the driver fatigue based on multiscale dynamic features, but camera-based techniques can’t perform in dark environments. Biomedical variables like respiration rate related to the nervous system may provide direct information of driver physiological conditions. Hence, they are extremely important to determine the drowsiness cycle and anticipate risky situations during driving [22,23,24]. In [25], the tracking and localization of cyclists using UWB technology is discussed, however, the researchers did not consider the crashes due to cars and other big vehicles and it has no method to detect the mobile phone usage or drowsiness detection of the drivers. Electroencephalogram (EEG) was used by researchers [18] to detect driver drowsiness, but in case of EEG measurement electrodes are used for the measurements which require physical contact with the person. The researchers in reference [19] used EEG, electrocochleogram (EOG), and electrocardiogram (ECG) signals for detection of driver drowsiness based on the physiological conditions of the driver, but this solution is really uncomfortable as the driver has to physically wear the devices. The ANSWatch device was used for detection of heart rate in reference [20], but it requires the driver to always wear the watch. Researchers in [21,22,23] also used EEG and/or ECG for physiological condition monitoring which is cumbersome for the driver. Vital sign measurement for drivers using radar can solve all the above problems at the same time. The radar operates robustly in the dark, and by measuring the vital signs in a noncontact manner without any inconvenience to the driver, an alarm can be generated in advance before the driver makes a mistake due to drowsiness.

In our work, we monitored the vital signs, i.e., respiration and heart rate of the driver inside a real car using IR-UWB radar. The UWB technology offer several benefits such as accurate ranging, robustness to multipath interference, better penetration and rejection of interferences [26,27]. Although there are algorithms in the literature [28,29,30,31] that deal with vital sign measurement, however, the conventional algorithms do not estimate the vital signs during the motion state. In our paper, we present a novel algorithm that can extract the vital signal from the radar signals reflected from the human body which are slightly distorted by the driver motions due to steering use or electronic and mechanical devices operation. The main concept of our vital sign estimation algorithm is that the vital signals are reflected from many points of the chest and abdominal area of the human sitting in front of the radar. Although the driving activity decreases the signal to noise ratio (SNR) of some part of the vital signal reflected from some area of the body, but still it doesn’t decrease the SNR of all the signals reflected from the body. Our goal is to search for those segments of the signals which have the best vital signs information and which are least distorted by the motion caused by driver activity. To this end, we first determined those signals which have better sinusoidal shapes and hence better vital signal information by using the sinusoidal fitting algorithm. Then we segment all those sinusoidal signals into sub-signal components and select those sub-signal components which have better correlation with the vital signal. In this way, we construct our desired vital signal from many signals reflected from the driver body. Then FFT algorithm is applied to the constructed vital signal to find the respiration and heart rate values.

Another main cause of collisions is mobile phone use during driving. Distracted driving caused due to the usage of cell phone (texting or speaking), is associated with 26% of all crashes and is increasing in frequency [32]. The mobile phone usage during driving results in restriction of sight; limiting drivers’ ability to monitor the road since their line of vision is focused on the handset [33]. It also reduces the concentration level and situational awareness [34,35,36]. Motivated by its impact on public safety and property, several state and federal governments have enacted regulations that prohibit driver mobile phone usage while driving [35]. In [36] a computer vision-based method for detection of driver cell phone usage by using a near infrared (NIR) camera system inside the car was presented. Smith et al. [37] have presented an algorithm for the detection of handset usage by the driver by analyzing images taken inside the car. Xu et al. [38] have proposed a machine-learning-based method for detecting driver cell phone usage using a camera system directed at the vehicle’s front windshield. These methods, however, are dependent on a camera, which may not provide good performance in dark environments. In our work, we proposed an algorithm to detect the use of mobile phones while driving by using IR-UWB radar that is unaffected by the light conditions. The proposed algorithm can accurately detect the use of mobile phones even in environments where driver's various movements or change of background objects occur inside the car. We propose a dual mode background subtraction algorithm using two different clutter signals for optimum detection, which is explained in Section 3.2.

The main contribution of our work is that we propose an algorithm using a single IR-UWB radar for monitoring the vital signs and detecting the mobile phone usage of the driver that can be used to develop technologies to prevent accidents caused by drowsiness driving and cell phone use. Our main purpose of the monitoring of driver’s vital signs is to provide a method for future researchers to use it for drowsiness detection so that the accidents due to drowsiness may be reduced. We propose an algorithm to accurately measure vital signs during the motion caused by the driving activities. We also suggest a way to detect the use of a cell phone that takes the driver's attention away. The proposed algorithm suggests a new method to optimally detect drivers’ cell phone usage considering various situations that can occur in the car. The technologies presented in this paper may become one of the technologies to save human life by being utilized in application technologies for preventing drowsiness driving and cell phone use during driving that are the root cause of many road crashes.

In Section 2 of the paper, the problems related to the previous methods for vital measurement and mobile phone detection are discussed. The signal preprocessing such as clutter removal is also discussed in Section 2. Section 3 is about our proposed algorithms for vital sign measurement during various driving conditions and phone detection in a specific area inside a car. Section 4 is related to the experimental results and in Section 5 the paper is concluded.

## 2. Problem Description and Signal Preprocessing

### 2.1. Problem Statement

In this paper, we cover two topics to prevent car crashes. One is to measure the driver's vital signs to prevent drowsy driving and the other is to detect the use of mobile phones to prevent accidents caused by their use while driving.

#### 2.1.1. Monitoring of Vital Signs

**Figure 1:**
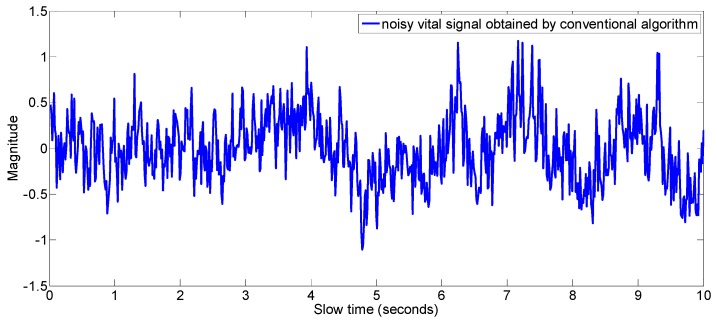
Noisy vital signal obtained by conventional algorithm during motion period.

Although there exist algorithms in the literature to measure the vital signs of a human [28,29,30,31], vital sign measurement during driving is a totally different problem as it involves different hand and body movements. The vital signs obtained during motion are deteriorated by the motion and vital signs can’t be measured from the signal. An example of such a signal is shown in Figure 1. The detection of the vital signs is difficult due to the movement of the driver as there is no trace of any periodicity in the received signal, which is a characteristic of vital signs. Researchers in [28] have discussed the problem of vital sign measurement of a non-stationary human, but the approach is very simple as they detect the motion of the body and lock the vital sign measurement until the body motion is stopped. In this work, however, we not only detect the body motion but also measure the vital signs during the motion period. The conventional method of extraction of vital signs at a fast time index of maximum variance doesn’t always hold true when random body movement is involved, therefore, we have used the data fitting method (see Section 3.1.1) to find those signals located at fast time indexes which gives better sinusoid fits. The vital signals are constructed from those better fit signals which exist even in presence of motion of some parts of the human body. After the vital signal construction, the conventional Fourier transform technique is applied to find the breathing and the heart rate.

#### 2.1.2. Detection of Phone Usage

A cellular phone are composed of electronic parts, PCB, and antenna. Their main components are metals such as copper, so cell phones have a very large radar cross-section (RCS). Although a large RCS makes it easy to detect a cell phone, there are some difficulties in detecting the use of a cell phone in the car. First, in the mobile phone sensing area of a car, various objects besides a mobile phone can appear. For example, several movements such as hand movements to press a button or grab beverage bottles to drink can occur, and the radar can detect it. In fact, these short time movements may be necessary to drive and those are not dangerous actions that distract the driver, therefore any detection algorithm should ignore them.

Secondly, in a really dangerous situation, like when the driver is staring at his cell phone for a while, there is little movement of the cell phone. If only the conventional background removal method [39] in Section 2.2 is used to detect the mobile phone in this situation, the mobile phone may be recognized as a background object and may not be detected. Considering the above two situations, an algorithm for detecting the use of mobile phones in a car should ignore very short time actions that are not dangerous to the driver and generate an alarm for any type of mobile phone use which is dangerous to the driver.

For a momentary movement that does not interfere with the driving, the alarm can be disabled by measuring the duration of the detection. In other words, we can ignore the detection of movement that appears for less than a certain time. In addition, to solve the problem that the signal reflected from the mobile phone is removed as background noise when there is almost no movement of the mobile phone, such as watching the screen quietly, we proposed a dual-mode background subtraction algorithm in Section 3.2. In this algorithm, background removal is performed in two ways, and mobile phone usage is detected by using two received signals obtained by each background removal method.

In the dual-mode background subtraction algorithm, immediately after the handset is detected, the clutter signal is not updated and the background is removed by using the clutter immediately before sensing the cell phone. In this way, it is possible to detect mobile phones with minimal movement. However, when the mobile phone is detected in this way, the mobile phone detection alarm will continue to be sounded when a new background object is placed in the detection area. To solve this problem, we have noted the difference between the received signal from the new background object and the received signal from usage of the mobile phone. There is a slight movement, even if mobile phone is held static like when just looking at a screen. Therefore, when the background is removed while updating the clutter signal, the received signal is small but is detected. On the other hand, the new background object has no motion, so if the background is removed while updating the clutter signal, the received signal is not detected at all. By using these differences, it is possible to distinguish a new background object from a mobile phone.

### 2.2. Signal Pre-Processing

The loopback filter is applied to remove the clutter from the raw signal [39]. The loopback filter is represented by the following equations:

(1) $$c_{k}\left(t\right)=\propto c_{k-1}\left(t\right)+\left(1-\propto \right)r_{k}\left(t\right)$$

(2) $$y_{k}\left(t\right)=r_{k}\left(t\right)-c_{k}\left(t\right)$$

**Figure 2:**
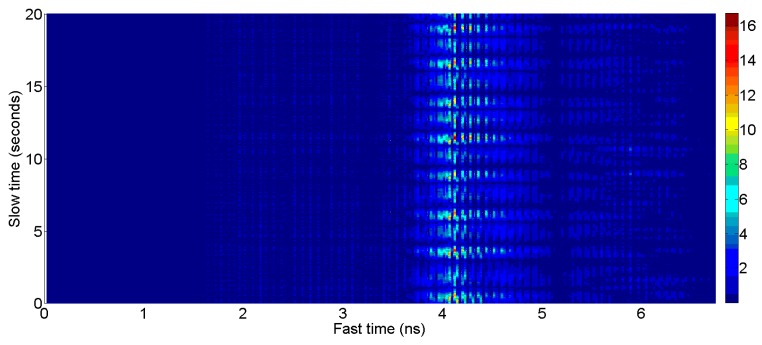
“Vital signal” vs slow time and fast time.

In Equations (1) and (2), the symbol “∝” is a constant used for weighting. The “∝” value in Equation (2) used for background subtraction was set to 0.97 for vital signs monitoring whereas for the mobile phone detection it was kept 0.8. A low alpha value means that the background removal rate is fast but vulnerable to noise, and if the alpha value is large, the background removal rate is slow but robust against noise. We have chosen the alpha value which is optimal for the corresponding application through several experiments. The symbol $r_{k}\left(t\right)$ show the received signal and symbol $c_{k}\left(t\right)$ shows the clutter signal, which is made until the *k*-th received sample. The background-subtracted signal is represented by the symbol$\;y_{k}\left(t\right)$. We need to store each filtered signal waveform and combine them into matrix W_mn_ of size “m×n”. The “m” represents the slow time length whereas the “*n*” represents the fast time length of the matrix. The value of “*n*” depends on the observation distance and has a value of 256 for our experiments (which means 1 meter observation distance) while “*m*” is a user choice and it depends on how long is the observation time. The concept of the slow time and fast time is shown in Figure 2.

Moreover, we only use specified areas for the detection of vital signs and phone detection by extracting only the fast time data of a specific area. The sensing area for phone detection has 25 cm range whereas sensing area for vital signs monitoring has a range of around 50 cm. This helps ignore environmental noise that comes from outside the sensing area. In other words, all the human activities that are out of this range are not taken into account during application of the vital sign measurement and the phone detection algorithm.

## 3. Proposed Algorithm

### 3.1. Vital Sign Measurement

**Figure 3:**
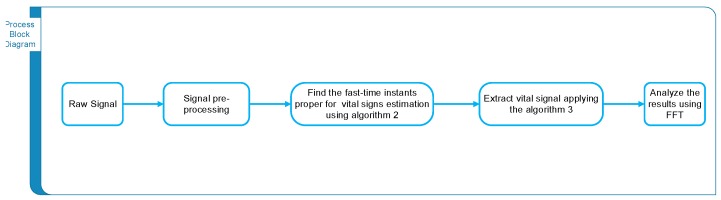
Process block diagram for extraction of driver vital signs.

The process block diagram for extracting the vital signs from the radar scans is shown in Figure 3. During driving, the hands continuously move because the driver has to control the steering, brakes and other devices inside the car. The conventional algorithm for vital sign measurement of a stationary human supposes that the biggest movement caused by our body is due to the vital signals of breathing and human heart, which is true for the case of stationary humans, but in case of body motions this is not always correct, so conventional algorithms for finding the breathing and heart rate will not provide accurate measurements in such conditions.

We observed that during driving, the major part of the motion exists in the upper part of the driver body and the lower part (abdominal area) is relatively stationary. Therefore, we first find which part of the body has motion due to the vital signals and which part of the body has motion due to random hand gestures, steering control or brake application. To this end, we use the sinusoidal data fitting algorithm, explained below.

#### 3.1.1. Extracting Receive Signals Which Have Useful Vital Sign Information

After we combine the waveforms into a matrix, we need to find which fast-time domain samples represent the sinusoidal motion, which is caused by the breathing and human heart motion. We use sinusoidal fitting algorithm (Algorithm 1).to show how much the received data fit into the sinusoid [40,41]. The input signal is s[n] and we want to estimate the frequency, amplitude and phase shift of this signal.

**Algorithm 1:** Sinusoidal data fitting algorithm for parameters estimationThe signal s[n] in its general form can be represented as sum of sinusoids as follows:(3)$$s\left[n\right]=\sum_{l=1}^{p}\left(\alpha_{l}\sin\left(\omega_{l}n+\varphi_{l}\right)+C_{l}\right)$$In Equation (3), $\alpha_{l}\;$is real valued constant describing the amplitude and $\omega_{l}\;$represent angular frequency, $\varphi_{l}\;$is the initial phase. The constant $C_{l}\;$shows the mean value other than zero. It is convenient to write the Equation (3) as follows.
(4)$$s\left[n\right]=\sum_{l=1}^{p}(A_{l}\cos\left(\omega_{l}n\right)+B_{l}\sin\left(\omega_{l}n\right)+C_{l})$$
It is convenient to use a vector representation of Equation (4). Let’s stack the samples in column vector as follows:
(5)$s=\overset{p}{\underset{l=1}{\sum }}H_{l}\theta_{l}$In the above Equation (5);
(6)$$H_{l}=\left[\begin{array}{c} \cos\left(\omega l\right)\;\;\sin\left(\omega l\right)\;1\\ \cos\left(2\omega l\right)\;\sin\left(2\omega l\right)\;1\\ .\\ .\\ .\\ \cos\left(N\omega l\right)\;\sin\left(N\omega l\right)\;1 \end{array}\right]\;\theta_{l}=\left[\begin{array}{c} A_{l}\\ B_{l}\\ C_{l} \end{array}\right]$$In our case, the model given by Equation (4) is simplified as; p=1 and C=0. Consider a signal *s*[*n*] with unknown parameters A,B,C and ω i.e., Equation (4) with p=1. The measured signal is then given by s[n] deteriorated by additive noise w[n] as shown in Equation (7):
(7)x[n]=s[n]+w[n] n=1,2,3, ….. , N
The noise is assumed zero mean white Gaussian noise. The estimation problem is to estimate the signal parameters using the measured samples of the input data x=[x(1), x(2), …. x(N)]. The probability density function (pdf)
(8)$$p\left(x;\theta ,\omega \right)=\frac{1}{\left(2\pi \sigma^{2}\right)}\exp\left[\frac{-1}{2\sigma^{2}}{\left(x-H\theta \right)}^{T}\left(x-H\theta \right)\right]$$
The above equation describes the probability per infinitesimal volume of receiving the data samples *x* given a set of parameters {θ,ω}.The maximum likelihood estimator (MLE) tries to maximize the pdf with respect to unknown parameters for given values of *x* and use those parameters as estimates i.e.,
(9)$\left[\hat{\theta },\hat{\omega }\right]=\arg_{\;\theta ,\omega }^{max}p\left(x;\theta ,\omega \right)$Finally, the estimate of θ is given by least-squares solution as follows
(10)$\hat{\theta }={\left(H^{T}H\right)}^{-1}H^{T}x\;$By using the least-squares solution, the MLE criterion function can be concentrated to one parameter as follows
(11)$g\left(w\right)=x^{T}H{\left(H^{T}H\right)}^{-1}H^{T}x$The frequency estimate is then obtained from maximizing the function g(w), that is (12)$\hat{\omega }={arg}_{\;\omega }^{max}g\left(w\right)$
The Equation (12) can be solved either by using iterative step method i.e., Gauss-Newton iteration [42] or by a non-linear search.

The input data used for sinusoidal fitting is the slow time domain data at each fast time index. The R-square value is used for finding the fit of the signal, which is defined as follows:

(13) $$R^{2}=1-\frac{\sum_{i=1}^{n}{\left(y_{i}-{\hat{y}}_{i}\right)}^{2}}{\sum_{i=1}^{n}{\left(y_{i}-\bar{y}\right)}^{2}}$$

**Figure 4:**
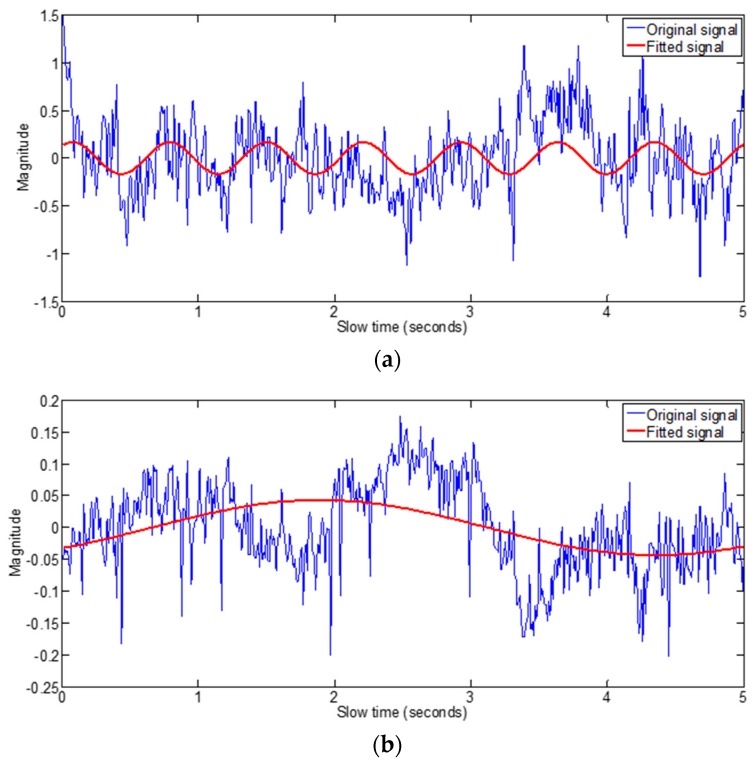

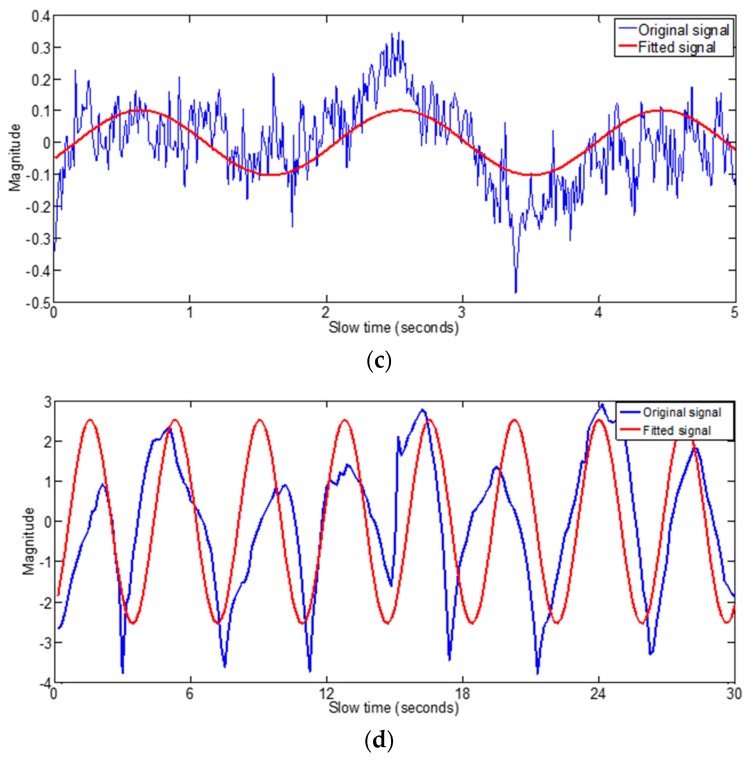
Data fitting algorithm results (**a**) fitting frequency higher than the respiration frequencies range (**b**) fitting frequency lower than the respiration frequencies range (**c**) fitting magnitude less than vital signal threshold (**d**) fitting signal for signal having information of vital signs.

**Figure 5:**
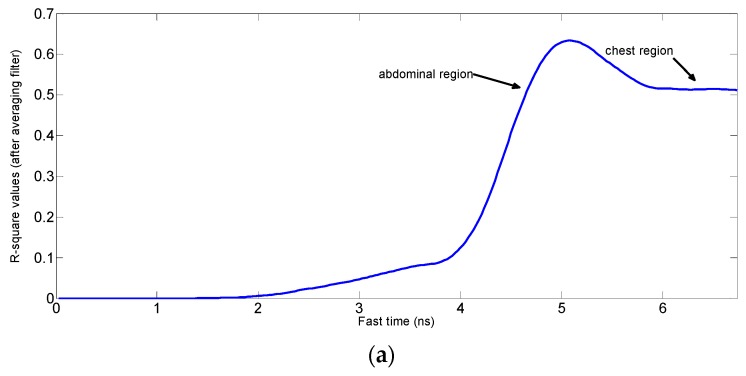

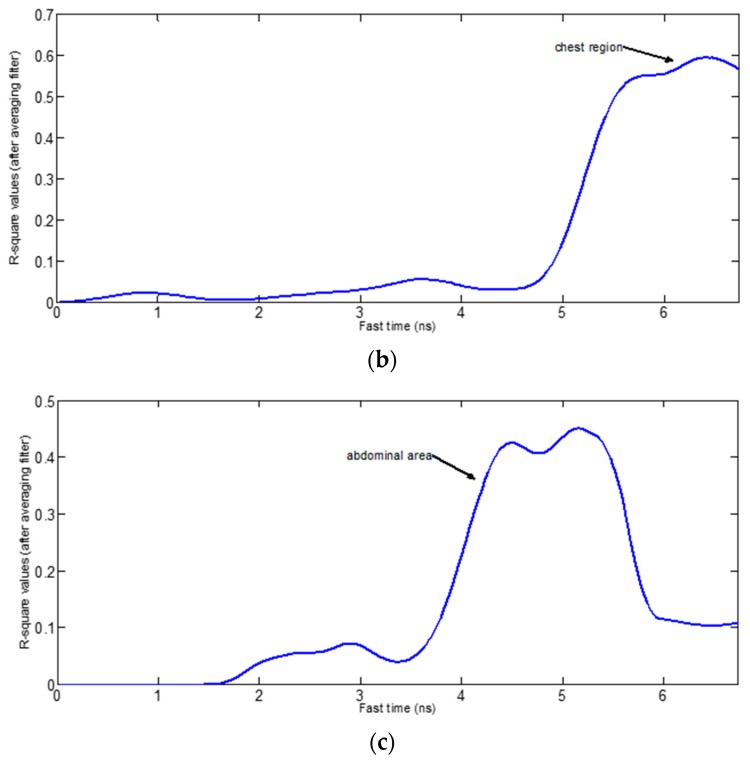
R square values while (**a**) stationary (**b**) moving hand near abdominal region (**c**) moving upper parts of body slightly.

In Equation (13), “$\hat{y}$” represent the estimated values of “y” by the fitting algorithm whereas “$\bar{y}$” shows the mean of “y” [43]. R square is a statistic that give information about the goodness of fit of a model. In general, a model fits the data well if the differences between the observed values and the model’s predicted values are small and unbiased. It determines how well the regression line or curve approximates the real data points. An R square value of 1 indicates that the regression line or curve perfectly fits the real data points. R-square can take on any value between 0 and 1, with a value closer to 1 indicating that a greater proportion of variance is accounted for by the model. For example, an R-square value of 0.73 means that the fit explains 73% of the total variation in the data about the average. The higher value of R square means that the sinusoidal regression model is more accurate and hence the motion is more sinusoidal and hence caused by the respiration and heart rate signals whereas the lower value of R square means non-sinusoidal motion caused by any random body/hand motion. We observed the R square values for all the fast time indexes. We have set the threshold value of 0.3 for finding the best fit sinusoids that contain useful information about the respiration and heart rate. If the value is kept too small then it may also select the signals that doesn’t have good vital signal information because they would not match sinusoidal data and if it is kept too high then we may not get any signal above the threshold because the vital signal is not perfectly sinusoidal and moreover, the movement of the body causes the signal to noise ratio of the vital signal to be very low. Therefore, we chose the optimal threshold value of 0.3 for finding the best fit signals. The detailed algorithm for finding the best sinusoid fits is given in Algorithm 2:
**Algorithm 2:** Selection of the fast time indexes that gives the best fit signalsFind the sinusoid fit as shown in Algorithm 1, which gives three parameters i.e., magnitude, frequency and phase shift of the sinusoid that best fits the signal.Find the R square values for the signals at each fast time index.If the fitting frequency of a signal is not in the range of the respiration frequencies i.e., 0.15 Hz to 0.5 Hz, as shown in Figure 4a,b then replace its R square value by zero.If the fitting sinusoid magnitude is less than a certain threshold value as shown in Figure 4c then replace its R square value by zero. Figure 4d shows the fitting for the signal that have the vital signs information as it has relatively higher magnitude and its frequency lies in the breathing frequencies range i.e., 0.15 Hz to 0.5 Hz.Apply the moving averaging filter to the resulting R square values as shown in Figure 5.Extract received signals with R values above threshold value.

Figure 5a shows the result of the fitting algorithm for the case when the person sitting in front of the radar is stationary. Figure 5b shows the R square values for the case when we move hand in front of the abdominal region, which results in lower R square values at the location of the abdominal region while the chest region signal gives better R square values. In experimental scenario for the Figure 5c, the upper parts of the body are moving due to hand gesture and/or steering motion exerted by the hands. From the comparison of these three figures, we know that the R square values decrease at the points (abdominal area in Figure 5b and chest area in Figure 5c due to the motion of the body parts, even though the variance of the signal increases. If we use the conventional algorithm in which the signal at the highest variance point is considered as vital signal, then it will result in wrong measurements when applied in a condition when the maximum variance is caused by the random body motion instead of the vital signs.

**Figure 6:**
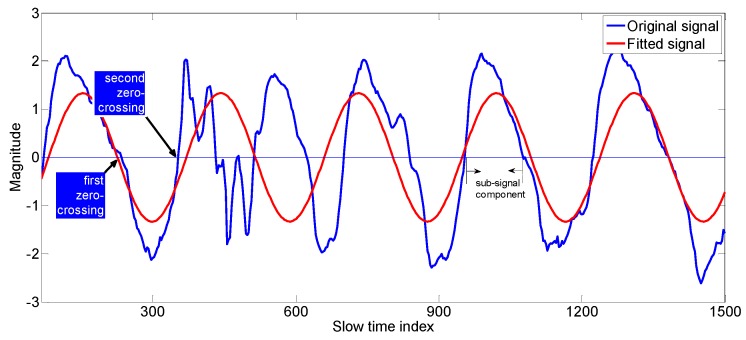
A best fit signal which has the corresponding r-square value of 0.46.

After finding the R square values of the signals in fast time index, we chose signals which have the good R-square values and ignore signals which have small R-square values. Figure 6 shows an example of the selected best fit signals which contain useful information about the vital sign signal. The reason that we expressed the slow time in term of index values rather than seconds is that Algorithm 3 uses the slow time index information for the construction of vital signal.

#### 3.1.2. Estimate of Breathing and Heart Rate through the Vital Signal Reconstruction

Now we have to construct the vital signal from the best-fit signals. First, we divide each signal into sub-signals based on the zero crossings as shown in Figure 6. A sub-signal component of a signal is defined as the signal samples between two consecutive zero crossing values. After dividing the best-fit signals into segments (sub-signal components), we constructed whole vital signal recursively from those segments using correlation concept as explained in Algorithm 3. For the sake of clarity, we have inserted some examples enclosed in the rectangular boxes inside the Algorithm 3.

We constructed the vital signal from the segmented good-fit signals by combining each sub-signal component to the previously constructed vital signal recursively and finding its autocorrelation. We chose the optimum sub-signal component that shows the highest autocorrelation. We have determined the autocorrelation of the signal after combining the new sub-signal-component with the previously constructed vital signal. If these signals have good correlation then the width of the autocorrelation (width_correlation) will be wider, otherwise the width of the autocorrelation will be narrower [28].

**Figure 7:**
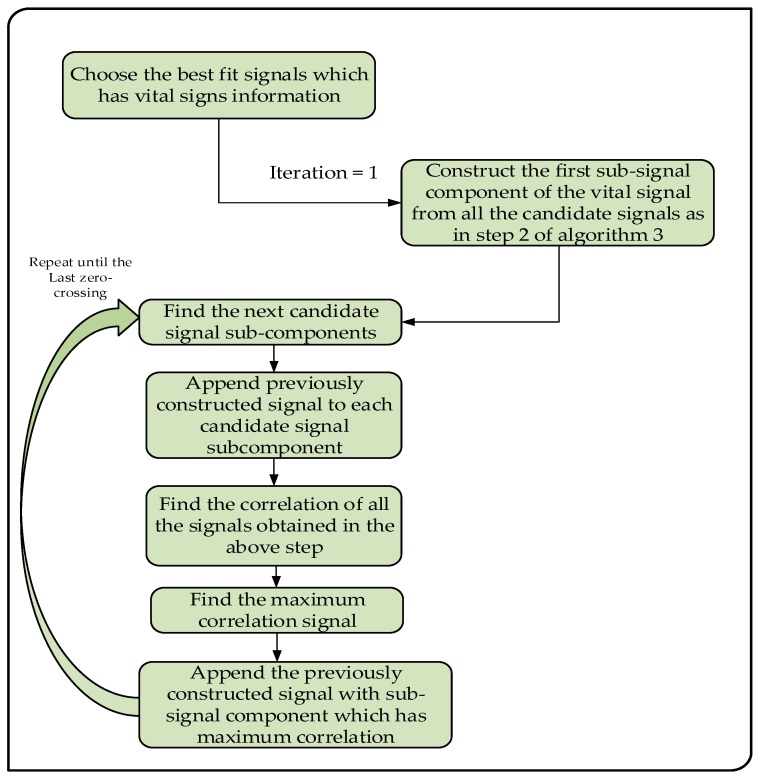
The vital signal construction from the candidate sub-signal components of the best fit signals.

In Figure 7, the big picture of the Algorithm 3 is explained by a diagram. The algorithm explains how to construct the vital signal from multiple best fit signals reflected from the body during motion of the driver.

The step by step explanation of the vital signal construction is explained by the following algorithm (Algorithm 3).

**Figure 8:**
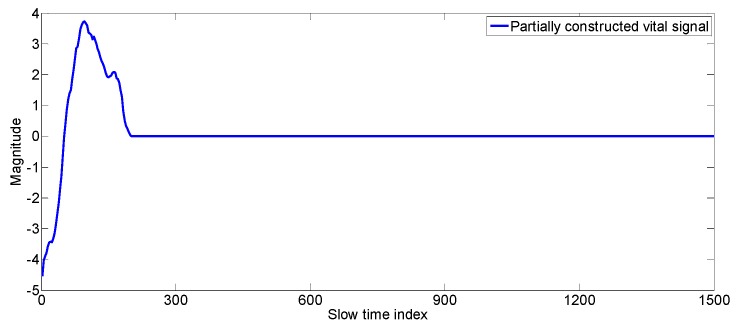
The first sub-signal component of vital_constructed_signal.

**Figure 9:**
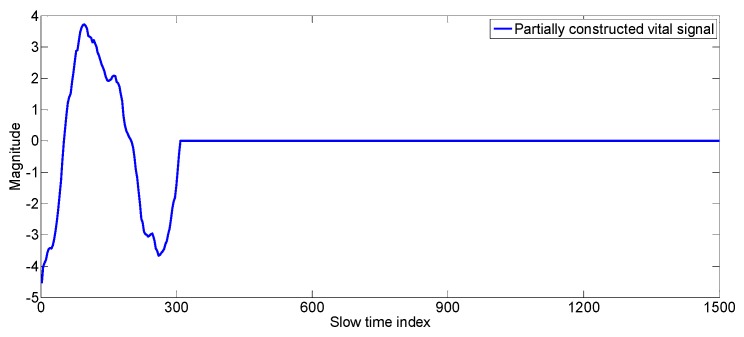
The constructed vital signal after second iteration.

**Figure 10:**
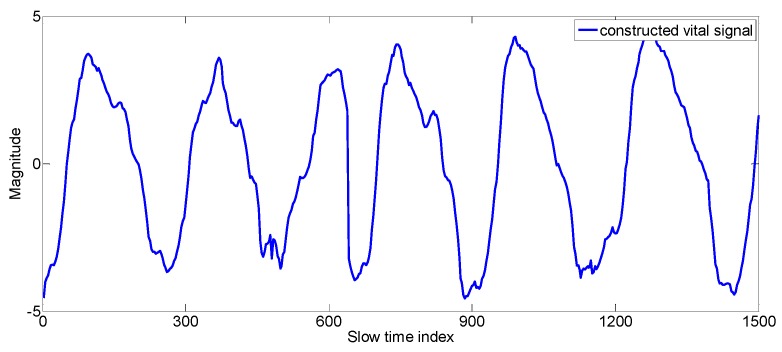
The final constructed vital signal.

**Algorithm 3:** Step by step construction of the vital signal from the best fit signals(1) Initialization Find “k” best-fit signals whose R-square values are above certain set threshold, say R-square_min = 0.3.The size of each best-fit signal is 256 samples. So, we have a matrix of $best\_fit_{matrix}\;\left(k\times 256\right).$ In our example, k = 9. (2) **for**
iterations=1:last_zero_crossing **If (iteration == 1)** **Then** Find the first zero-crossing fast time index (zero_crossings) of all the “k” best-fit signals. And find the sub-signal component, which has the maximum value of zero_crossings For our example, first_zero_crossings are found to be as follows:
first zero_crossings=[234 231 223 231 230 235 224 220];
max_zero_index=max (zero_crossings) = 235;
$$k\_max\_crossing=argmax_{k}(zero\_crossings)=6$$
Construct the vital signal according to the following expression, the result is shown in Figure 8.
constructed_vital_signal(1:max_zero_index)=best_fit_matrix(k_max_crossing,1:max_zero_index)
For our example; Figure 8 show the construction of first sub-signal component of the vital signal: constructed_vital_signal(1:235)=best_fit_matrix(6,1:235);
**Else if (iteration > 1)** Find **the next** zero-crossing fast time index (zero_crossings) of all the “k” best-fit signals **for**
i=1:k Append the $i^{th}$ sub-signal component to the previously constructed vital signal. Find the correlation of the resulting signal
$$correlation\left(i\right)=autocorr([\mathrm{constructed}\_\mathrm{vital}\_\mathrm{signal};\;i^{\mathrm{th}}\;\mathrm{sub}\_\mathrm{signal}\_\mathrm{component}]);$$
**end**
max(width_correlation(i)); i=1:k
Append that sub-signal component to the previously constructed vital signal, which has maximum correlation as found by the above expression.For our example, when iteration = 2: second zero_crossings = [354   359   351   344   347   360   354   351];
After we appended each sub-component to the previously constructed vital signal and found the correlation of each signal, it was found to be:
correlation =[17.1  13.3  18.3  9.3  16.2  23.8  18.2  22.8];
$$k_{max_{corr}}=argmax_{k}(correlation)=6$$
$$\max\_corr\_index=argmax_{zero\_crossings\;}\left(correlation\right)=360$$
Here the 6th signal sub-component shown the maximum correlation with the previously constructed signal. Now we append the next sub-signal component of the 6th signal to the previously constructed vital signal to find the constructed_vital_signal as follows in Figure 9.
constructed_vital_signal(end:max_corr_index)=best_fit(k_max_corr,end:max_corr_index)
where end=length(previous constructed vital signal)+1 For our example:
constructed_vital_signal (236:360) = best_fit_matrix (6, 236:360);
(3) **Check**, if: there are further zero crossings, then go to step **2** **Else**; stop the loop; **Save**; the constructed_vital_signalThe final vital signal obtained is given in the Figure 10. **end**

In Algorithm 3, the vital signal is constructed from the sub-signal components of best-fit signals. Now we have to extract the breathing and heart values from the constructed vital signal using the Fast Fourier transform (FFT). The size of FFT used in the evaluation of the frequency domain signal was 2^15^ and the slow time sampling frequency was around 110 samples/s. The frequency resolution thus obtained was 0.2014/min. The respiration rate is extracted from the FFT signal by finding the location of the maximum peak of the spectrum as discussed in detail in the reference [28].

### 3.2. Dual-Mode Background Subtraction Algorithm for Phone Detection

**Figure 11:**
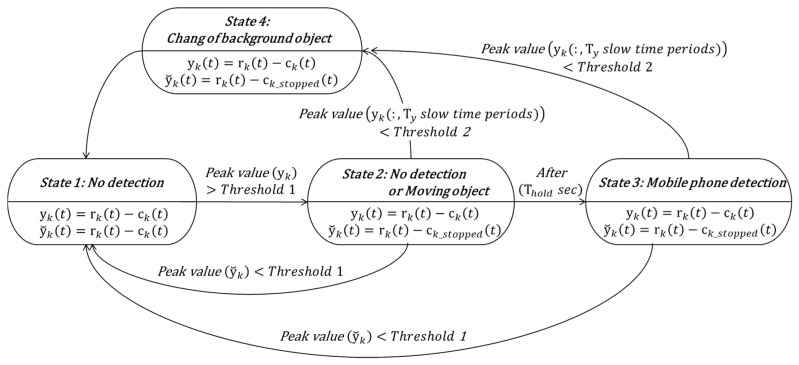
State diagram of dual-mode background subtraction algorithm for phone usage detection.

We proposed a dual-mode background subtraction algorithm to detect the driver’s use of the mobile phone accurately and robustly. Figure 11 shows a diagram for the proposed algorithm. State 1 indicates that the magnitude of the reflected signal exceeding the threshold is not detected in the radar detection area. The received signal is obtained by using the background subtraction method in Section 2.2. If the peak value of the received signal exceeds threshold 1, a transition from state 1 to state 2 occurs. In Equation (14), $c_{k\_stopped}\left(t\right)$ is the clutter signal immediately before state 1 → state 2 transition. And $k_{transient}$ is the slow time index at transition from state 1 to state 2. Parameter $k_{margin}$ is the slow time margin for transition:

(14) $$c_{k\_stopped}\left(t\right)=\propto c_{k_{transient}-k_{margin}-1}\left(t\right)+\left(1-\propto \right)r_{k_{transient}-k_{margin}}\left(t\right)$$

State 2 indicates a state in which a signal exceeding a threshold value 1 is detected by an instantly high noise or a moving object such as a hand gesture. In state 2, two received signals are obtained by a dual mode background subtraction method using different clutters. The first is the signal $y_{k}\left(t\right)$ obtained by using the background substation method in Section 2.2 and the second is the signal ${\overset{˘}{y}}_{k}\left(t\right)$ obtained by using $c_{k\_stopped}\left(t\right)$ clutter as shown in the following Equation (15):

(15) $${\overset{˘}{y}}_{k}\left(t\right)=r_{k}\left(t\right)-c_{k\_stopped}\left(t\right)$$

If it is an instantaneous moving object, the maximum value of ${\overset{˘}{y}}_{k}\left(t\right)$ will be smaller than threshold 1, which means state 2 → state 1 transition. If a new background object appears or an existing background object disappears, the maximum value of ${\overset{˘}{y}}_{k}\left(t\right)$ is still larger than the threshold 1, but the state 2 → state 4 → state 1 transition will occur because the maximum value of $y_{k}\left(t\right)$ in the $T_{y}$ slow time interval becomes smaller than the threshold 2. If the driver's mobile phone is detected, the transition from state 2 to state 3 occurs because the maximum value of $y_{k}\left(t\right)$ in the $T_{y}$ slow time interval is greater than threshold 2 and the maximum value of ${\overset{˘}{y}}_{k}\left(t\right)$ is greater than threshold 1 for a certain period of time ($T_{hold})$. State 3 indicates a state in which the use of the mobile phone is detected. If the maximum value of ${\overset{˘}{y}}_{k}\left(t\right)$ becomes smaller than threshold 1, a transition occurs to state 3 → state 1, which means that the use of mobile phone is stopped. Or when the maximum value of $y_{k}\left(t\right)$ in the $T_{y}$ slow time interval becomes smaller than threshold 2, a transition occurs to state 3 → state 4→ state1, which means that a new background object appears or that the existing background object disappears.

**Figure 12:**
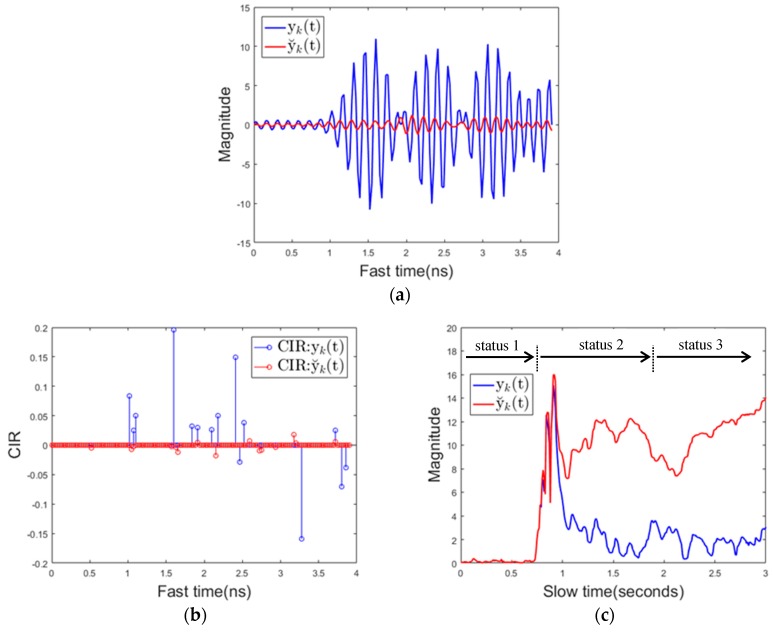
Signal characteristics when using a mobile phone (**a**) received signal over fast time (**b**) channel impulse response (**c**) the maximum value of the received signal over slow time.

Figure 12a below shows the magnitude of the received signal over fast time when the driver uses the mobile phone. It is shown that the magnitude of the reflected signal is much larger when using the proposed background removal method ${\overset{˘}{y}}_{k}\left(t\right)$ than the conventional background removal method$\;y_{k}\left(t\right)$. This is because, in the conventional method, the mobile phone is regarded as the background, and the signal reflected from the mobile phone is attenuated.

**Figure 13:**
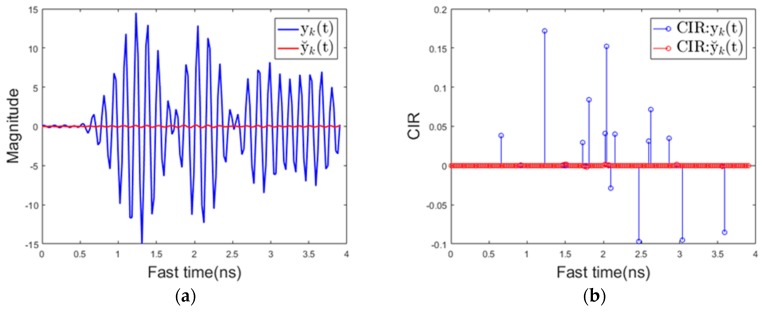
Signal characteristics when there is a background change (**a**) received signal over fast time (**b**) channel impulse response.

Figure 12b shows the channel impulse response (CIR). The CIR was estimated using the clean algorithm in [44]. This also shows that the CIR of ${\overset{˘}{y}}_{k}\left(t\right)$ is much larger than the CIR of $y_{k}\left(t\right)$ for the above reasons. Figure 12c shows the maximum value of the received signal over slow time in the same situation. At about 0.7 s, the mobile phone entered the sensing area, which caused both $y_{k}\left(t\right)$ and ${\overset{˘}{y}}_{k}\left(t\right)$ to increase in magnitude. At this point, the algorithm transitions from state1 to state2. At about 1 s, mobile phone almost becomes stationary except some minor motions due to texting or touching the screen, and after that point, the magnitude of ${\overset{˘}{y}}_{k}\left(t\right)$ still remains a large value. $y_{k}\left(t\right)$ represents a relatively small value because the mobile phone is recognized as the background. The mobile phone is continuously used in the detection area, and therefore, the maximum value of ${\overset{˘}{y}}_{k}\left(t\right)$ and the maximum value of $y_{k}\left(t\right)$ in the $T_{y}$ slow time interval are both larger than the respective threshold values, and thus the state transitions from state 2 to state 3 (mobile phone detection) occurs at about 1.9 s. In addition to the signal characteristics for cell phone use described here, the signal characteristics for various types of driver behavior are specified in the results section. Figure 13a,b below shows the magnitude of the reflected signal over fast time and its CIR when there is a background change. $y_{k}\left(t\right)$ represents the magnitude of a noise level while the magnitude of ${\overset{˘}{y}}_{k}\left(t\right)$ represent a large value. This characteristics of $y_{k}\left(t\right)$ can be used to distinguish background changes from using mobile phones with minor motion.

## 4. Results and Discussion

### 4.1. Experimental Setup and Reference Data Measurements

In our experiments, we used the commercially available single-chip IR-UWB radar transceiver NVA6201 made by NOVELDA (Novelda AS, Kviteseid, Norway). The radar has a center frequency of 6.8 GHz, a bandwidth of 2.3 GHz, and a transmission output power of −53 dBm/Hz. The pulse repetition frequency is 100 MHz and the slow time sampling frequency (measurement rate) is 110 samples/s. The radar transceiver supports staggered pulse repetition frequency (PRF), a transmission process in which the time between each coherent pulse transmission is patterned and slightly changed. This function extends the maximum unambiguous range (MUR) by making it possible to clearly distinguish the return pulses corresponding to the transmission pulses among many return pulses. In practice, the radar transceiver has a range of almost 10 m and the maximum range is determined by the SNR. The operating temperature of the radar transceiver chip is −40 to 80 degrees, which ensures stable operation under any environment.

**Figure 14:**
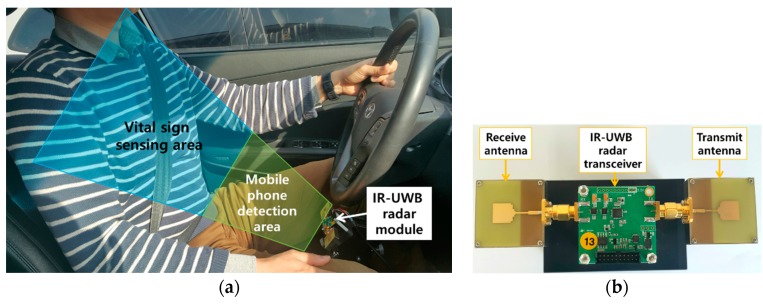
(**a**) Experimental setup inside the car (**b**) IR-UWB radar module.

The experimental setup of the radar inside the car is shown in Figure 14a. The sensing area is divided into two parts. The region which is near to the radar module is the phone detection region because in this region a driver usually positions his/her phone while reading messages or other mobile phone related activity which can cause distraction of the driver from his primary task of monitoring the road. The region right after the mobile sensing region is the vital sign detection region. It covers the whole front body area of the driver. Figure 14b shows the structure of the IR-UWB radar module. The radar module consists of a radar transceiver, a transmit antenna and a receive antenna. A patch antenna is used, and the antenna and transceiver board are connected by a SubMiniature version A (SMA) connector.

**Figure 15:**
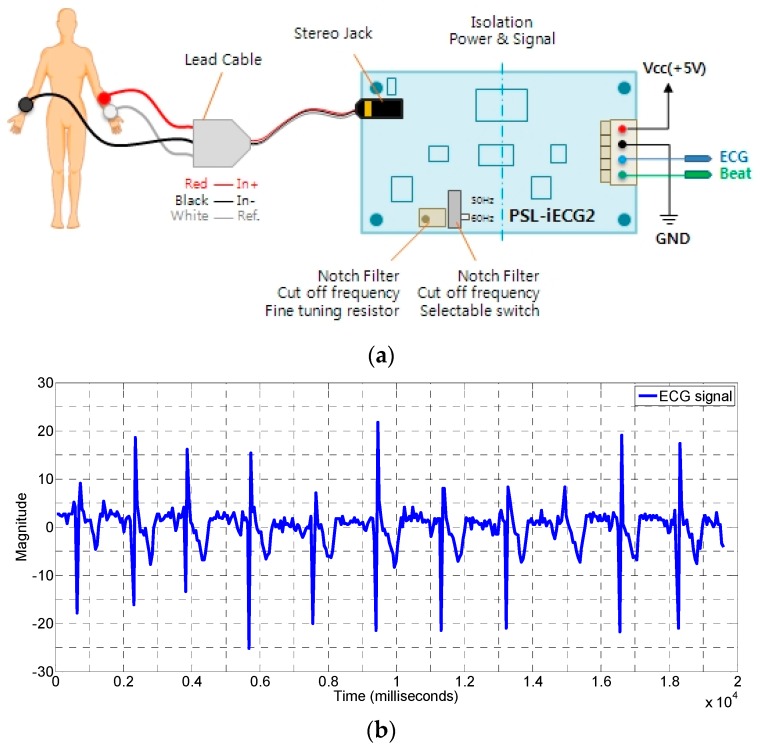
(**a**) Measurement setup for the ECG signal acquisition; (**b**) The signal obtained from the ECG sensor module PSL-iECG2 (measurement reference device).

In order to verify the results of our proposed algorithm for the heart rate measurement, we have to compare it some benchmark device such as ECG sensor. We used the ECG sensor module PSL-iECG2 which require only 5 V input voltage (Vcc) and the current consumption is below 50 mA. The amplification is 750 V/V. It uses a selectable 50 or 60 Hz enabled by the notch filter switch. The measurement setup for the connection of the electrodes to the human hands is illustrated in Figure 15a [45].

The sample output of the ECG signal is shown in Figure 15b. We also used some reference measurement for the validity of our respiration rate value by the proposed algorithm. In many studies, respiration rate is measured by wearing a band on the belly or chest [46], but in case of our experiments, if we cover the chest or belly with a band, the measurements are affected because the reflected signal from the belly or chest is distorted by the band. Therefore, we used nasal breath sound recordings from a smartphone as a reference for respiration rate measurements. As in [47], the reference respiration rate can be measured accurately with the errors less than 1% for all breathing ranges.

### 4.2. Respiration & Heart Rate Results

First, we give an example to show the performance of our algorithm as compared to a conventional algorithm such as the one described in reference [28] during the motion period. After the example, we verify the proposed algorithm for different types of motions related to the driving activities and measure the vital signs during the motion period and the results are given in Table 1 and Table 2. In order to show that our algorithm is repeatable, we tested it for five different human subjects and calculated the difference between the reference results and the results from our proposed algorithm and summarized the results in Table 3.

**Figure 16:**
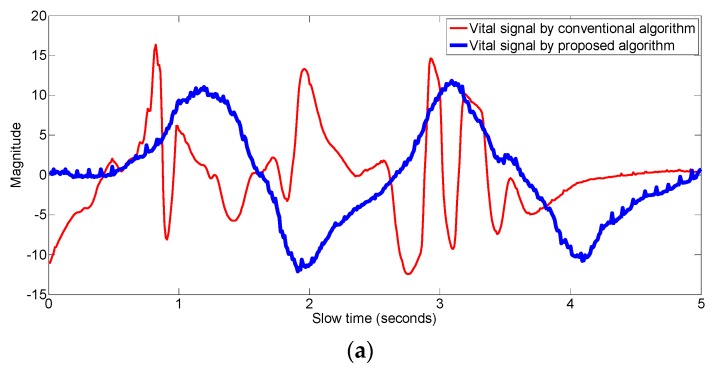

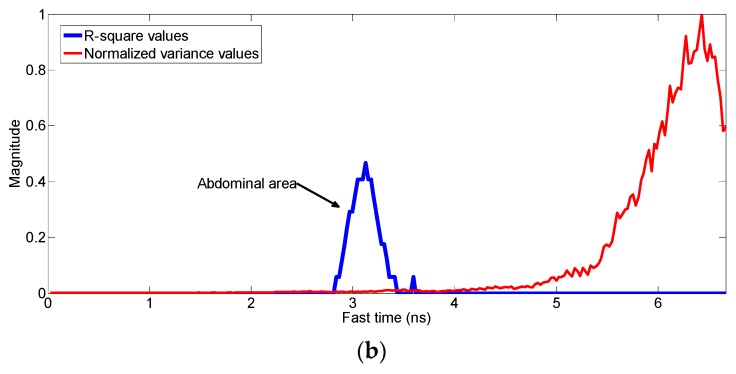
(**a**) Comparison of the vital signal obtained from the conventional vs proposed algorithm while the human is slightly moving (**b**) R-square and normalized variance values at each fast-time index during motion state.

In order to show that the proposed algorithm extract the vital signal even if there is some motion of the body, we made an experiment as follows. In the experiment, slight motion of the upper part of the body is made. The vital signals that are obtained by the conventional algorithm as well as by the proposed algorithm are shown in Figure 16.

**Figure 17:**
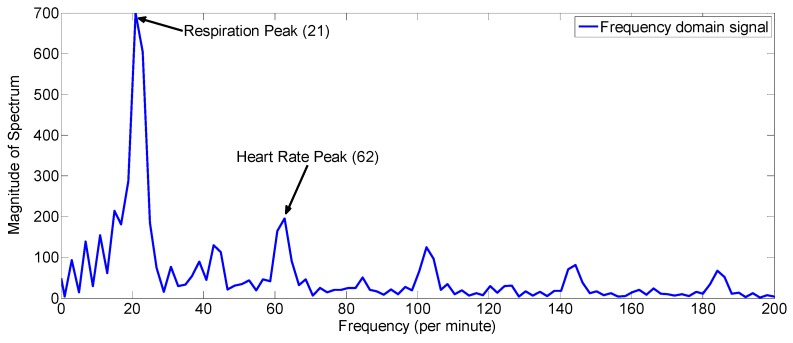
Spectrum obtained by applying FFT algorithm to the signal obtained by proposed algorithm in Figure 16a.

As is shown in Figure 16, in a state of slight motion, conventional algorithm looks for the signal at fast time index having maximum variance as the vital signal, however, the motion is not due to the respiration and heartbeat motion but it is due to the random body motion as shown by the values of the R-square in blue-color line in Figure 16b. The vital signal obtained by the conventional algorithm in Figure 16a does not have any trace of periodicity, whereas the respiration signal obtained by the proposed algorithm in this example has good periodic pattern and it captures the vital signal information. In Figure 17, the frequency domain signal is shown. The highest peak i.e., the peak at 21 cycles/minutes represent the breathing frequency whereas the second highest peak in the heart frequency range i.e., at 62 cycles per minute represent the heart rate of the human.

Driving involve different kinds of motion of hand, head and body such as looking at the side mirror to check the road behind, or picking a glass in front of the driver or hand motion associated with the steering operation. We also taken into account, the external effects like turning the car, applying the brakes due to external factor and accelerating the car. The conventional algorithms for vital signs measurement didn’t specifically deal with vital signs measurement during such motions. We measured the vital signs during the motion activities and summarized the results in Table 1 and Table 2 as follows. The values of the measurements are rounded to the nearest integer values.

Table 1 show the results for the estimated respiration rate for different body motion states and it also shows the number of best fit signals reflected from body for each motion case. In case of body motion related to driving such as in the Table 1, our proposed algorithm can find some good-fit signals which can be used to extract the vital signal and hence it can give good measurement results.

We also measured the heart rate during different body movements which may be made by a driver. The results are summarized in Table 2 and compared with the reference heart rate measurements. The values of the measurements are rounded off to the nearest integer values.

**Figure 18:**
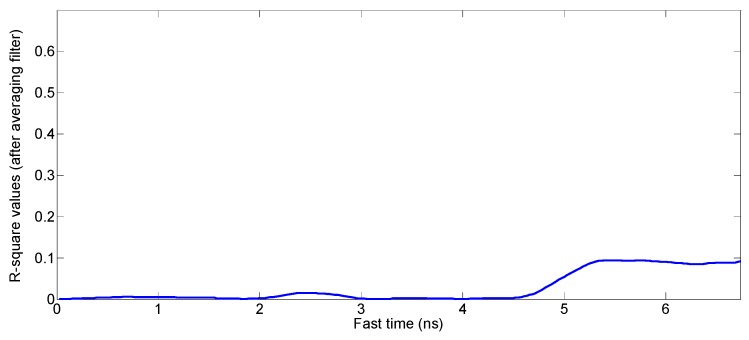
The R-square values in case of huge body movements (all the values are below the threshold value of 0.3).

The above Table 2 shows the results of the heart rate measurements during the body movements of a driver. The results are related to the general movements of human as well as specific motions related to driving activities. The results in Table 2 show that the algorithm works well under various motion conditions. During huge body movements, which rarely happen during driving activities such as turning the car speedily and passing at high speed over a speed bump, there is no signal which can fit a sinusoidal signal (the R-square values of the signals obtained during huge motion of the car are shown in Figure 18), which clearly indicates that all the R-square values are below the threshold value for the best fit signals i.e., 0.3. Usually, during driving the most of motions are made by hands/arms, shoulder and chest so vital signs measurements by our proposed algorithm is suitable for the driving conditions.

In order to show the validation of the proposed algorithm, we used different human subjects for our experiments. The average error (difference between the reference value and the estimated value) results of the vital signs for five humans are shown below in Table 3. The people involved in these experiments were healthy and aged between 24–38 years old.

The results in Table 3 show that the algorithm works correctly and robustly regardless of the person.

### 4.3. Mobile Phone Detection Results

In order to verify the proposed cell phone detection algorithm, several experiments were conducted. As shown in the diagram in Figure 11, the signals received in the sensing area are processed in two ways to remove the background and two post-processed signals are obtained. The use of the mobile phone is detected while comparing the maximum values of the processed signals with the threshold value. Threshold 1 is 3.0 and threshold 2 is 0.8. $T_{hold}$ is 1.2 s and $T_{y}$ is 1.0 s. These configuration parameters have been optimized so that the alarms are generated and released naturally and the detection error is minimized through several experiments.

**Figure 19:**
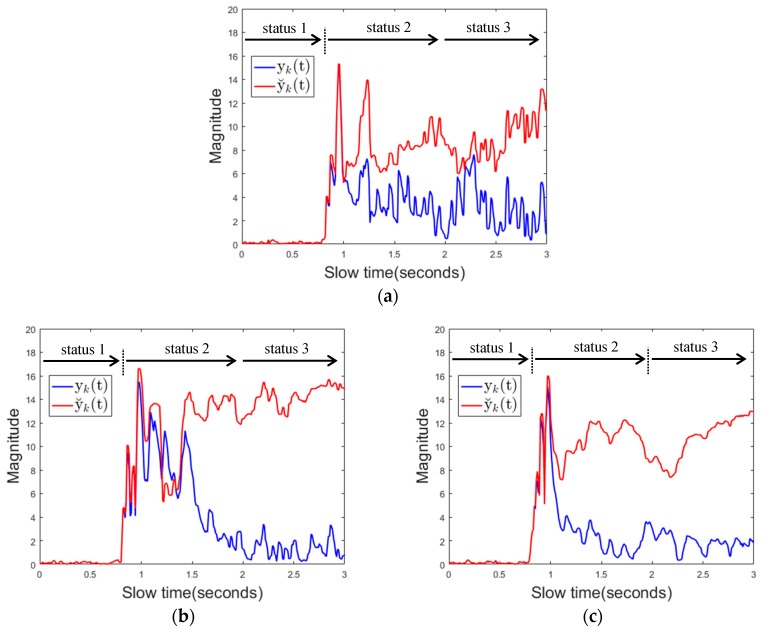
The maximum value of the received signal over slow time when using a mobile phone (**a**) texting (**b**) scrolling, touching (**c**) viewing.

The first is an experiment to determine whether the proposed algorithm correctly recognizes that the mobile phone is in use when the driver uses the mobile phone in various ways. Figure 19 shows the characteristics of the received signal over time for each case. Both $y_{k}\left(t\right)$ and ${\overset{˘}{y}}_{k}\left(t\right)$ are consistently higher than thresholds. In this case, the state transition in the algorithm occurs in order state 1 (no detection) → state 2 (no detection or moving object) → state 3 (mobile phone detection).

**Figure 20:**
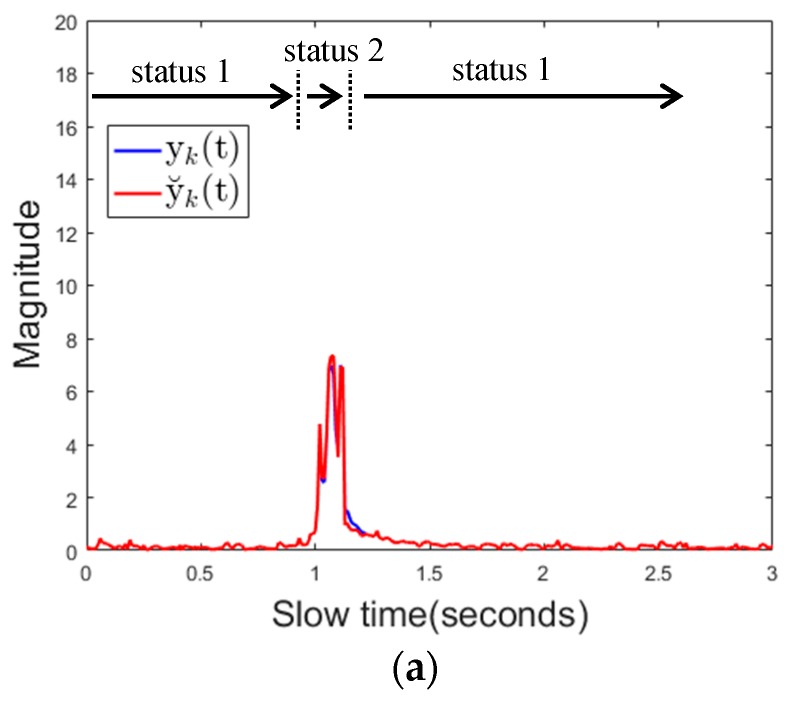

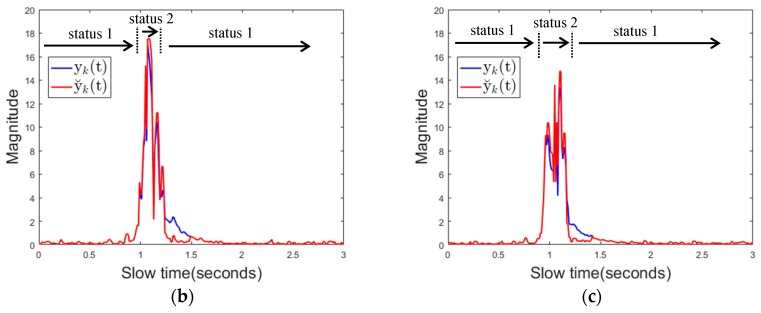
The maximum value of the received signal over slow time with momentary movements. (**a**) moving hand (**b**) moving water bottle (**c**) moving cell phone.

Next is whether the algorithm will detect moving objects as a mobile phone or not when there is a moving object in the sensing area for a while. Figure 20 shows the characteristics of the received signal over time for each case. It can be seen that the magnitudes of the signals $y_{k}\left(t\right)$ and ${\overset{˘}{y}}_{k}\left(t\right)$ instantaneously become larger than the thresholds and the magnitude becomes smaller as the object moves out of the sensing area. In this case, state transitions occur as state 1 (no detection) →state 2 (no detection or moving object) → state 1 (no detection).

**Figure 21:**
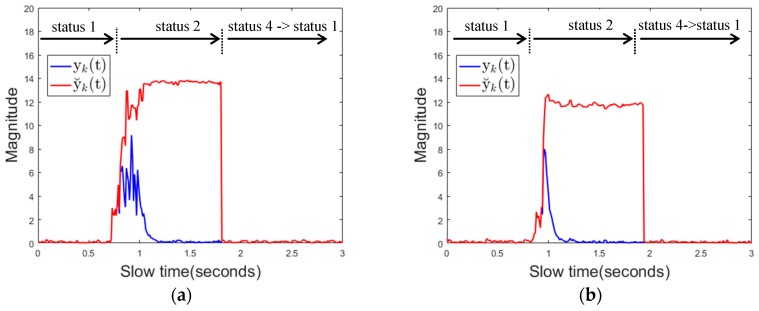
The maximum value of the received over slow time when there is a background change (**a**) water bottle (appearing) (**b**) water bottle (disappearing)

Next is, if there is a change of the background object in the sensing area, whether the proposed algorithm detects this change as the use of the mobile phone or not. Figure 21 shows the characteristics of the reflected signal over time for each case.

The magnitude of the signal ${\overset{˘}{y}}_{k}\left(t\right)$ is consistently greater than the threshold 1, but the magnitude of $y_{k}\left(t\right)$ is only increased when the object enters the sensing area, and soon it become less than the threshold 2. In this case, the state transition occurs as state 1 (no detection) → state 2 (no detection or moving object) → state 4 (change of background object) → state 1 (no detection) or as state 1 (no detection) → state 2 (no detection or moving object) → state 3 (mobile phone detection) → state 4 (change of background object) → state 1 (no detection). Table 4 shows the detection results when 5 people repeated 50 repetitive actions for each experimental case. There is no miss detection at all and it also shows that there is no false alarm when there is no cell phone use. Moreover, the proposed algorithm does not generate false alarms for instantaneous hand gestures or some moving objects and even if a new background object appears or disappears.

## 5. Conclusions

We have presented techniques based on IR-UWB radar that can be helpful in developing methods for preventing car crashes from happening. The monitoring of vital signs related to drowsiness driving and the detection of mobile phone usage by the driver are the two main purposes of this paper. Firstly, we presented how to extract vital signs signal from the measurements during motion of the body due to driving activity. We found the fast time locations which have better information about the vital signal of the driver. We divided the signals at those locations into segments and constructed the vital signal based on the correlation concept. After construction of the vital signal, an FFT algorithm was applied and the respiration and heart rate were found. Another objective of this paper was to detect the use of mobile phones while driving. The proposed algorithm distinguishes and detects the driver’s cell phone use from various other actions or changes inside the car using the dual mode background subtraction method. Experimental results shown that the proposed mobile phone detection algorithm works perfectly in most of the scenarios that can occur in a car. If these technologies are combined, then it may be very useful for avoiding the car crashes due to drowsiness or mobile phone usage of drivers.

## Figures and Tables

**Table 1:** Experimental results for breathing rate for different motion conditions.

Movement Case		No. of Best-Fit Columns above R-Square Threshold (0.3)	Reference Measurement (Breathing Rate)	Estimated Value (Breathing Rate)
stationary		19	18	18
general movements	hand motion near body	8	18	19
	shoulder motion for steering	4	18	16
	gesture made by hand away from body	11	18	18
	lips motion during speaking	14	18	18
specific driving movements	head movement to watch the mirror of the car	7	18	18
	turning the car	4	18	20
	applying the brakes	7	18	17
	accelerating the car	10	18	18

**Table 2:** Experimental results for heart rate measurements for different motion conditions.

Movement Case		No. of Best-Fit Columns above R-Square Threshold (0.3)	Reference Measurement (Heart Rate)	Estimated Value (Heart Rate)
stationary		17	72	72
general movements	hand motion near body	9	72	71
	gesture made by hand away from body	11	73	72
	lips motion during speaking	12	72	73
specific driving movements	head movement to watch the mirror of the car	8	72	74
	turning the car	5	72	75
	applying the brakes	7	74	71
	accelerating the car	12	72	72

**Table 3:** Average error for respiration and heart rate of different human subjects (five humans).

Movement Case		Average Error (Bpm) (Respiration Rate)	Average Error (Bpm) (Heart Rate)
stationary		almost zero	almost zero
general movements	hand motion near body	0.7	1.1
	gesture made by hand away from body	0.3	0.9
	lips motion during speaking	0.2	0.6
specific driving movements	lead movement to watch the mirror of the car	0.3	2.0
	turning the car	1.2	2.5
	applying the brakes	0.5	1.7
	accelerating the car	0.4	1.3

**Table 4:** Mobile phone detection results.

Object	Detail Action	Detection Rate	False Alarm Rate
**mobile phone**	texting	100% ^(1)^	0% ^(2)^
	scrolling, touching	100% ^(1)^	
	viewing	100% ^(1)^	
**moving object for a moment**	moving hand	N/A	0% ^(3)^
	moving water bottle		0% ^(3)^
	moving cell phone		0% ^(3)^
**change of background**	water bottle (appearing)		0% ^(4)^
	water bottle (disappearing)		0% ^(4)^

^(1)^ The probability of being recognized by a mobile phone when using a real mobile phone. ^(2)^ The probability of recognizing the use of a mobile phone even though there is nothing in the detection area. ^(3)^ The probability of detecting an instantly moving object as the use of mobile phone. ^(4)^ The probability of detecting change of background as the use of mobile phone.
